# Celiac disease and Down syndrome mortality: a nationwide cohort study

**DOI:** 10.1186/s12887-017-0801-4

**Published:** 2017-01-31

**Authors:** Jonas F. Ludvigsson, Benjamin Lebwohl, Peter H. R. Green, Wendy K. Chung, Karl Mårild

**Affiliations:** 1 Department of Medical Epidemiology and Biostatistics, Karolinska Institutet, 171 77 Stockholm, Sweden; 2 Department of Pediatrics, Örebro University Hospital, Örebro University, Örebro, Sweden; 3 Celiac Disease Center, Department of Medicine, Columbia University College of Physicians and Surgeons, New York, NY USA; 4 Division of Molecular Genetics, Department of Pediatrics, Columbia University Medical Center, New York, NY USA; 5 Division of Epidemiology, Norwegian Institute of Public Health, Oslo, Norway

**Keywords:** Celiac, Coeliac, Death, Down syndrome

## Abstract

**Background:**

Individuals with Down syndrome (DS) have increased mortality and are also at increased risk of celiac disease (CD). It is unknown if CD influences mortality in DS. In this study we examined the risk of death in individuals with DS according to celiac status.

**Methods:**

In this nationwide population-based cohort study, we first identified individuals with CD (diagnosed 1969–2008) through small intestinal biopsy report data showing villous atrophy (Marsh stage III) from Sweden’s 28 pathology departments. Celiac individuals were then matched with up to five reference individuals from the general population. In these cohorts we identified individuals with DS using International Classification of Disease codes (ICD) registered in the Swedish Patient Register (includes inpatients and hospital-based outpatients), the Medical Birth Register, and the Register of Congenital Malformations. Of 29,096 individuals with CD, 201 (0.7%) had DS compared to 124 of the 144,522 reference individuals (0.09%). Data on mortality were obtained from the Swedish Cause of Death Registry. Hazard ratios (HRs) for death were calculated using Cox regression.

**Results:**

During follow-up, there were seven deaths among individuals with DS and CD (7/201, 3.5%) as compared with 14 deaths among DS individuals without a record of CD (14/124, 11.3%). Adjusting for potential confounders, CD did not influence the risk of death in DS (HR = 1.36; 95%CI = 0.33–5.59). Cardiovascular death occurred in two individuals with CD and three individuals without CD, while death from malignancy occurred in one individual with CD and two individuals without CD.

**Conclusion:**

While both DS and CD have been linked to increased risk of death, this study found no excess mortality in DS patients with a concurrent diagnosis of CD, however confidence intervals were wide.

**Electronic supplementary material:**

The online version of this article (doi:10.1186/s12887-017-0801-4) contains supplementary material, which is available to authorized users.

## Background

Down syndrome (DS, trisomy 21) is the most frequent chromosomal abnormality in the US, with 1 in about 700 liveborn children having DS [1]. Individuals with DS are at increased risk of death, both overall and specifically from congenital heart defects, dementia and leukemia as compared with individuals without DS [2, 3]. However, in many economically developed countries there has in the last decades been markedly improved survival in DS patients. In the US, the median survival increased from 25 years in 1983 to 49 years in 1997 [3]. This increased life expectancy in DS patients is believed in large part to be attributed to advances in medical management including early detection and treatment of comorbid conditions, such as improved provision of cardiac surgery for children with DS with congenital heart defects [3, 4].

Celiac disease (CD) is an immune-mediated disorder that occurs in about 1–2% of the Western population and which is triggered by exposure to gluten in genetically susceptible individuals [5]. CD has been linked to an increased risk of death in most [6], but not all, studies [7]. Common causes of death include cardiovascular disease and cancer [8]. CD has also been associated with a number of disorders, including type 1 diabetes [9] and mood disorders [10, 11].

Since the 1970s there have been reports on an association between DS and CD, and the largest study to date in this field [12] found a 6-fold increased risk of biopsy-verified CD in individuals with DS. Today, the leading medical guidelines on DS and CD recommend CD screening in individuals with DS [13–15]. As more and more individuals with DS are diagnosed with CD, we were motivated to examine if concurrent CD influences the risk of death in DS.

We hypothesized that CD would be associated with an increased risk of death in patients with DS.

## Methods

In this nationwide population-based cohort study, we first identified individuals with CD through small intestinal biopsy report data. Government agencies matched each individual with CD with up to five controls from the general population. In these cohorts we then identified individuals with DS using data from national healthcare registries.

Through Cox regression we calculated Hazard ratios (HRs) for death in individuals with DS and concurrent CD as compared with DS individuals without a CD diagnosis.

### Study participants

In 2006–2008 we collected small intestinal biopsy reports from all of Sweden’s 28 pathology departments (biopsies per se had been carried out in 1969–2008). Data were retrieved by local IT-personnel and included information on personal identity number [16], biopsy date, topography (duodenum or jejunum) and morphology according to the Swedish SnoMed classification (see our earlier paper for a detailed description [17]). We defined CD as having villous atrophy (VA, in this study equivalent to Marsh stage III [18]). While a positive serology was not a prerequisite for the celiac diagnosis, in a random sample of individuals, some 88% were positive for CD serology at time of biopsy [17]. After the removal of duplicates and data irregularities, we had data on 29,096 individuals with CD (identical to the individuals in our earlier study on mortality in CD [8]).

### Handling of matching

Individuals with CD were then matched for sex, age, county and year of biopsy with up to five controls from the Swedish Total Population Register [19]. We did not restrict controls to individuals with a negative biopsy since that would select a control population with more comorbidity than the general population since healthy people do not undergo routine biopsy. This matching was originally carried out to construct two cohorts (one with CD and one without CD). We then identified all individuals with DS from the CD cohort and from the control cohort. Since DS occurs in less than one percent of the population, we did not keep our initial matched strata in the statistical analyses in the current study but instead adjusted for potential confounders.

### Down syndrome

We defined DS through any of the following ICD (International Classification of Diseases) codes (ICD-8: 759,30 and 310–315,51; ICD-9: 758A; ICD-10: Q90) in any of the registers: the Swedish National Patient Register [20], the Medical Birth Register [21], and the Register of Congenital Malformations [22].

The Swedish National Patient Register started in 1964, became nationwide in 1987 and includes hospital-based outpatient care since 2001 [20]. The positive predictive value of most diagnoses is 85–95%, and it is estimated to cover >99% of all non-psychiatric health care in Sweden. The Swedish Medical Birth Register started in 1973 and includes > 98% of all births in Sweden. The National Register of Congenital Malformations collects data on severe malformations and chromosomal abnormalities since 1964. According to the National Board of Health and Welfare, holder of the national health registers in Sweden, the combined data from multiple registers enable a close to complete identification of individuals with DS, including all three genetic variants of DS: trisomy 21 caused by meiotic non-disjunction, mosaicism and translocation. The Patient Register allows identification of DS also for individuals born before 1973. A DS diagnosis can be recorded at any age when a patient seeks healthcare (i.e. not only at birth).

### Mortality

We used data from the Swedish Cause of Death Register to ascertain overall mortality and cause-specific mortality.

### Covariates

Data on socioeconomic status (according to the European Socioeconomic Classification: six categories with missing data fit into a separate category [23]) and education level (four a priori categories) [23] were obtained from the government agency Statistics Sweden. Other covariates included country of birth (Nordic versus not Nordic), year of CD diagnosis (1989 and earlier, 1990–1999, 2000 and later) and age at CD diagnosis (0–1.99; 2–9.99; 10–39.99; 40+ years). Data on type 1 diabetes were obtained from the Swedish Patient Register (see Additional file 1).

### Statistics

We used Cox regression with years since study entry as the time metric to estimate the HR for death in patients with DS. We used a staggered entry statistical approach since the original study sample included individuals at date of biopsy and matched controls who were alive at the corresponding date. Follow-up then ended with date of death, emigration or end of study (Dec 31, 2009). As described above we broke the matching of our original study sample since DS only occurs in a very small proportion of participants (and to find several DS patients in a stratum consisting of one CD patient and five controls is extremely unlikely), and instead adjusted for potential confounders. Therefore, HRs were adjusted for sex, age at celiac diagnosis (or corresponding date of inclusion as a control), calendar period at first biopsy with CD, socioeconomic status, level of education, country of birth and type 1 diabetes.

Pre-planned analyses included HRs according to calendar period at first celiac biopsy (1969–1989; 1990–1999; 2000–2009), sex and age. We present stratified HRs for individuals with CD diagnosed in childhood (0–15 years) versus adulthood (16+ years).

A power calculation showed that our study could detect (at 0.05 significance and 80% power) a 2.26-fold increased risk of death in patients with both DS and CD compared to those with DS only.

We used SPSS 22 (SPSS, Inc. Chicago, IL, USA) and R survival package for the analyses. Statistical significance was defined as 95% confidence intervals (CIs) for risk estimates not including one and a two-sided *p*-value < 0.05.

### Ethics

This study was approved by the Regional Ethical Review Board in Stockholm, Sweden (June 4, 2006; 2006/633-31/4). No individual consent was needed since data were strictly register-based [24]. Data linkages with national registers were initially performed by the government agencies Statistics Sweden and the National Board of Health and Welfare.

## Results

Of 29,096 individuals with CD, 201 had DS (1 in 144). Similarly, 124 of the 144,522 population-based controls had DS (1 in 1165). Hence, this study included 325 individuals with a diagnosis of DS (Table 1). Only 5 individuals with DS were born outside the Nordic countries. More than 40% of individuals with CD were diagnosed after the year 2000 (Table 1). The median year of CD diagnosis was 1995.

**Table 1:** Characteristics of patients with Down syndrome

	Celiac disease	Not Celiac disease
Total	201	124
Year of birth (median; range)	1958; 1942–1994	1943; 1921–1991
Year of death (median; range)	2002; 1997–2011	2005; 1999–2010
Females (%)	97 (48.3)	82 (66.1)
Males (%)	104 (51.7)	42 (33.9)
Not Nordic country of birth (%)	3 (1.5)	2 (1.6)
Type 1 diabetes (%)	1 (4.8)	12 (3.9)
Age, at study entry (years)		
0–1.99 (%)	45 (22.4)	27 (21.8)
2–9.99 (%)	71 (35.3)	19 (15.3)
10–39.9 (%)	71 (35.3)	47 (37.9)
≥ 40 (%)	14 (7.0)	31 (25.0)
Calendar year at study entry		
− 1989 (%)	10 (5.0)	28 (22.6)
1990–99 (%)	102 (50.7)	44 (35.5)
2000– (%)	89 (44.3)	52 (41.9)

During follow-up, there were seven deaths among individuals with DS and concurrent CD (7/201, 3.5%) as compared with 14 deaths among DS individuals without a record of CD (14/124, 11.3%). Of these seven individuals with CD, four were females and three were males. Cardiovascular death occurred in two individuals with CD and three individuals without CD, while death from malignancy occurred in one individual with CD and two individuals without CD. The median age at death was 51 years in patients with CD, and 61 years in those without CD.

Concurrent CD was not associated with risk of death in DS (adjusted HR [aHR] = 1.36; 95%CI = 0.33–5.59). The neutral aHR despite the larger proportion of deaths in DS individuals without CD was due to a skewed age distribution among DS patients. Some 25% of DS patients without CD entered the study at the age of 40 years or later, compared to only 7% of DS patients with concurrent CD (Table 1).

While none of the risk estimates were statistically significant, the aHRs for death were low in DS individuals with a diagnosis of CD in childhood (0.18; 95%CI = 0.02–1.85), and high in those with an adult CD diagnosis (5.78; 0.76–43.9) (p for interaction between age and CD was 0.018).

Since there was only 1 death in DS individuals with CD diagnosed before 1989, and 1 death from 2000 and onwards (5 died between 1990 and 1999), we did not examine the risk of death according to CD status for individual calendar periods.

Individual causes of death are presented in Table 2.

**Table 2:** Causes of death among patients with Down syndrome

	Celiac disease	Not celiac disease
Total	7	14
Chronic leukemia	1	1
Dementia	1	
Heart disease or atherosclerosis	2	3
Liver cirrhosis	1	
Down syndrome^a^	2	4
Neurodevelopmental disorder		1
Brain tumor		1
Pneumonia due to aspiration		1
Influenza		1
Renal failure		1
Accident		1

^a^Diagnostic codes for underlying syndromes are often recorded as the underlying cause of death in the Swedish Cause of Death Register. In Sweden, however, the causes of death in individuals with DS have not been validated

## Discussion

In a nationwide cohort of more than 29,000 individuals with biopsy-verified CD we identified approximately 200 individuals with a concurrent diagnosis of DS. Through linkage with national register data we were able to examine the role of CD in mortality in DS. While our study had limited power, our data argue against CD influencing mortality risk in DS.

DS has large phenotypic variation, with some features occurring in all individuals with DS, including learning disability, craniofacial stigmata and hypotonia in early infancy [25]. DS is frequently associated with congenital heart defects, early-onset dementia and childhood leukemia. Apart from CD, other autoimmune diseases such as type 1 diabetes or thyroid disorders are common in DS patients [26, 27]. To identify such associated diseases, Swedish children with DS are followed at pediatric outpatient clinics at least once per year, while adult DS patients often are seen biannually in primary care. Overall, health supervision of individuals with DS has improved over the last decades, being one factor for the improved life expectancy seen in the population with DS [4]. Still, earlier population-based data have found that individuals with DS have an overall 11-fold increased risk of death, with an excess cause-specific mortality from infectious diseases, heart disease and dementia etc [2]. However, the potential influence of concurrent CD on DS mortality has previously not been examined.

The pathogenetic link between DS and CD is not fully understood. In general, the genetic susceptibility to CD is believed to be shared between human leukocyte antigen (HLA) genotypes on chromosome 6p21 and a large number of genes outside the HLA loci [28]. However, because individuals with DS have a similar distribution of HLA genotypes as the general population [28] candidate genes in DS have been searched among immune-related non-HLA loci. Two of the suggested candidate genes (interferon receptor 1 [*IFNAR1*] and interferon receptor 2 [*IFNAR2*]), indeed located on chromosome 21, encodes the receptor to the pro-inflammatory cytokine interferon-alpha, which is believed to play an important role in the intestinal immune response in CD [29].

This study has some strengths and limitations. We used three different independent national registers to identify individuals with DS. Together, these registers should have high sensitivity for DS. Although the accuracy of the DS diagnosis per se has not been examined in the Swedish national registers, it should be high, considering that the positive predictive value for other diagnoses in the Swedish National Patient Register is usually 85–95%. During follow-up, 21 individuals with DS died, and among the causes of death were typical causes in patients with DS (such as heart disease, dementia, and chronic leukemia), although numbers were too small to run cause-specific HRs. While the HR for death in DS individuals with an adult diagnosis of CD was not statistically significant (HR = 5.78), this finding may suggest that DS individuals with long-standing undiagnosed CD may be a group at increased risk. However, insufficient statistical power (there were only 21 deaths among individuals with DS) meant an excess risk could not be formally confirmed.

We used small intestinal biopsy data from all 28 pathology departments in Sweden to identify CD [17]. During the study period, more than 95% of all gastroenterologists and pediatricians performed a small intestinal biopsy before a CD diagnosis [17], and hence the use of pathology data should yield a high sensitivity for a CD diagnosis. Similarly, the specificity for CD should be high, since other causes of villous atrophy than CD are rare in Sweden. In a manual review of pathology reports in more than 1500 patient charts, the most common comorbidity mentioned was inflammatory bowel disease (Crohn’s disease or ulcerative colitis) which occurred in 0.3% of those with villous atrophy [17]. Biopsy reports were based on an average of 3 tissue specimen [30], which should detect 95% of all villous atrophy. When Swedish pathologists examined blinded samples of villous atrophy, they accurately graded 90% of these as villous atrophy. While we did not have data on symptoms in all patients with CD, in a subset of individuals undergoing patient chart validation (*n* = 118), 79% had gastrointestinal symptoms at diagnosis [17]. This is consistent with symptom data of other series of celiac patients [31].

This nationwide study included more than 200 individuals with DS and CD. Still, given the relative rarity of DS and concurrent CD we cannot rule out that we have failed to observe a true difference in excess mortality due to lack of study power. An alternative explanation is that the small excess mortality in CD is difficult to detect in DS patients who already have a high relative risk of death. Another limitation of this study includes our lack of data on dietary adherence in celiac patients with DS hence this study does not examine the role of a gluten-free diet in DS. However, a recent study by our group found that mucosal healing (often a strong indicator of dietary adherence) did not influence mortality after CD diagnosis [32].

Regrettably, we also lacked data on other clinical characteristics in order to decipher whether CD was diagnosed due to symptomatic disease or as a result of CD screening. During the time of study (up to 2008), there were in Sweden no national guidelines for CD screening in DS, and clinicians were instead advised to have a high awareness of CD- related manifestations and, if present, offer serological testing. Therefore, we cannot rule out some degree of misclassification of CD (i.e. false-negative) if not all patients with DS were screened for CD. The current Swedish medical care program for DS recommends repeated CD screening in children, but not adults, with DS. This explains the lower age in DS patients with CD (as opposed to those with only DS), since general screening of DS patients have been carried out especially in later years and in younger individuals. This is also why the adjusted relative risk for death was neutral despite a much lower absolute prevalence of death in DS + CD patients ((3.5%) compared to patients with DS only (11.3%).

## Conclusion

In conclusion, we found no excess mortality in DS patients with a concurrent diagnosis of CD. CD does not seem to be a significant risk factor for death in patients with DS.

## Additional file

Additional file 1: Appendix. Description: contains data on histopathology classification used in this study, and a list of international classification of disease (ICD) codes to identify type 1 diabetes. (DOC 41 kb)

## Abbreviations

CD: Celiac disease
CI: Confidence Interval
DS: Down syndrome
HLA: Human leukocyte antigen
HR: Hazard ratio
ICD: International Classification of Disease (codes)
VA: Villous atrophy
