# Supplementary material for: Celiac disease and Down syndrome mortality: a nationwide cohort study
Source: BMC Pediatr. 2017 Jan 31;17:41. doi: 10.1186/s12887-017-0801-4 (PMC5282819; doi:10.1186/s12887-017-0801-4)
Supplement: Additional file 1: — Appendix. Description: contains data on histopathology classification used in this study, and a list of international classification of disease (ICD) codes to identify type 1 diabetes. (DOC 41 kb) [file 12887_2017_801_MOESM1_ESM.doc]

**APPENDIX**

**Table - Small intestinal histopathology classifications – a comparison**

| ***Classification used in this project*** | ***Normal*** | ***Inflammation*** | | ***Villous atrophy*** | | |
| --- | --- | --- | --- | --- | --- | --- |
| Marsh Classification* | Type 0 | Type 1 | Type 2 | Type 3a | Type 3b | Type 3c |
| Marsh  Description | Pre-infitrative | Infiltrative | Infiltrative-hyperplastic | Flat destructive | | |
| Corazza et al  (*ref A*) | - | Grade A | | Grade B1 | | Grade B2 |
| SnoMed Codes | M0010, M0011 | M40000, M41000, M42000, M43000, M47000, M47170 | | M58,  D6218,  M58005 | M58,  D6218,  M58006 | M58,  D6218,  M58007 |
| KVAST/Alexander classification | I  Normal | II  Intraepithelial lymphocytosis (IEL)# | | III  Partial VA | IV  Subtotal VA | IV  Total VA |
|  |  |  |  |  |  |  |
| *Characteristics* |  |  |  |  |  |  |
| Villous atrophy | - | - | - | + | ++ | ++ |
| IEL# | - | + | + | + | + | + |
| Crypt hyperplasia | - | - | + | + | ++ | ++ |

*We have not included Marsh type 4 in this classification since such lesions are very rare (*ref B*) and cannot be identified through SnoMed Codes.

# Increased intraepithelial lymphocyte count (often >30/100 epithelial cells).

KVAST: Kvalitets- och Standardiseringskommittén (English: Committee for Quality and Standardization).

Ref A: 1. Corazza GR, Villanacci V, Zambelli C, et al. Comparison of the interobserver reproducibility with different histologic criteria used in celiac disease. Clin Gastroenterol Hepatol 2007;5:838-43.

International classification of disease codes used for *Diabetes mellitus, type 1*:

ICD7: 260; ICD8: 250; ICD9: 250; ICD-10: E10.

Since the Swedish ICD system did not distinguish between type 1 and type 2 diabetes until ICD-10 we defined type 1 diabetes as having a diagnosis of diabetes ≤age 30 years of age.
